# Aryl Hydrocarbon Receptor Is Required for Fasting-Induced Improvement of Gut Barrier Integrity in *Caenorhabditis elegans*

**DOI:** 10.3390/antiox14080905

**Published:** 2025-07-24

**Authors:** Junjie Sun, Yuseok Moon

**Affiliations:** 1 Laboratory of Food Systems Analytics, Department of Data Science, School of Data Science, Pusan National University, Busan 46241, Republic of Korea; erwang19525252@gmail.com; 2 Department of Foodtech and PNU-Korea Maritime Institute (KMI) Collaborative Research Center, Pusan National University, Busan 46241, Republic of Korea; 3 Department of Convergence Medical Sciences, Biomedical Research Institute, Pusan National University, Yangsan 50612, Republic of Korea

**Keywords:** *Caenorhabditis elegans*, aryl hydrocarbon receptor, calorie restriction, gut barrier, mitochondrial oxidative stress

## Abstract

The intestinal barrier governs organismal health through nutrient absorption, microbial homeostasis, and immune surveillance. While calorie restriction (CR) enhances metabolic health, the molecular mechanisms underlying its beneficial effects on gut integrity remain unclear. Here, we demonstrate that the aryl hydrocarbon receptor (AHR), a conserved xenobiotic sensor and metabolic regulator, is essential for CR-mediated improvements in intestinal function. Using *Caenorhabditis elegans* (*C. elegans*), we subjected wild-type (N2) and AHR-deficient strains (CZ2485 and ZG24) to ad libitum feeding (AL), intermittent fasting (IF), or complete food deprivation (FD). In wild-type animals, intermittent fasting markedly reduced intestinal permeability and bacterial burden while enhancing mitochondrial function and reducing reactive oxygen species. Complete food deprivation conferred modest benefits. Remarkably, these protective effects were severely compromised in AHR mutants, which exhibited increased gut leakage, bacterial colonization, and mitochondrial oxidative stress under fasting conditions. These findings establish AHR as a critical mediator of fasting-induced intestinal resilience, revealing a previously unrecognized regulatory axis linking metabolic sensing to gut barrier homeostasis. Our work illuminates fundamental mechanisms through which calorie restriction promotes gastrointestinal health and identifies AHR-dependent pathways as promising therapeutic targets for metabolic and inflammatory distress affecting the gut–systemic interface.

## 1. Introduction

The gastrointestinal tract is a critical interface between the external environment and the internal milieu, playing essential roles in nutrient absorption, immune surveillance, and microbiota regulation. Maintaining the integrity of the intestinal barrier is vital for overall health, and its disruption has been associated with a wide range of age-related diseases, including metabolic syndrome, chronic inflammation, and neurodegenerative disorders [1,2,3]. One of the key contributors to intestinal barrier dysfunction is oxidative stress, which impairs epithelial tight junctions and promotes increased gut permeability [4,5].

In recent years, calorie restriction such as IF and fasting-mimicking diets have received considerable attention for their ability to enhance metabolic resilience and extend lifespan [6,7,8]. IF, in particular, has been shown to reduce inflammation, improve redox homeostasis, and preserve intestinal barrier function in both animal models and humans [9,10]. However, the underlying molecular mechanisms that mediate these fasting-induced intestinal benefits remain incompletely understood.

The nematode *C*. *elegans* has emerged as a powerful model system for investigating gut physiology and aging. It shares many conserved signaling pathways with mammals, including those involved in oxidative stress responses and epithelial maintenance [11,12]. Importantly, intestinal barrier integrity is increasingly recognized as a key determinant of systemic metabolic health and aging. Loss of gut integrity can lead to increased translocation of microbial-derived endotoxins and pro-inflammatory molecules into the systemic circulation, triggering low-grade chronic inflammation, a hallmark of metabolic disorders and age-related decline [13,14]. Conversely, a preserved gut barrier helps reduce systemic oxidative stress, modulates host metabolism, and promotes healthy longevity.

Among the evolutionarily conserved regulators of stress and barrier function, the AHR stands out as a ligand-activated transcription factor that modulates xenobiotic metabolism, immune homeostasis, and epithelial integrity in mammals [15,16]. While AHR is well studied in vertebrate barrier tissues, its role in the context of dietary interventions such as fasting remains poorly understood. In *C. elegans*, the AHR homolog (*ahr-1*) is primarily expressed in neurons and has been implicated in developmental processes and lifespan regulation [17,18], but its function in intestinal protection under metabolic stress is still unclear.

In this study, we investigated how different dietary regimens, including AL, IF, or FD, influence intestinal barrier function and mitochondrial activity in wild-type *C. elegans* and two AHR mutants, CZ2485 and ZG24. By employing a combination of imaging-based bioassays in worms, we aimed to determine whether AHR is required for the beneficial effects of fasting on gut integrity and to explore the potential link between nutritional intervention, redox regulation, and gut resilience.

## 2. Materials and Methods

### 2.1. C. elegans Strains and Maintenance

Wild-type N2 and AHR mutant strains (CZ2485 and ZG24) were obtained from the Caenorhabditis Genetics Center (CGC, University of Minnesota, USA). Both CZ2485 and ZG24 are *ahr-1* loss-of-function mutant strains, but they differ in their specific alleles: CZ2485 carries the ju145 allele, which is a deletion mutation resulting in complete loss of AHR-1 function, whereas ZG24 carries the *ia3* allele, a point mutation that similarly abolishes AHR-1 activity. Importantly, the ZG24 strain is homozygous viable yet exhibits distinct neuronal defects, particularly affecting GABAergic neuron differentiation, which highlights the functional consequences of the *ahr-1* mutation [19,20]. By including both independently generated mutant strains with different types of molecular lesions, we sought to ensure that the phenotypes observed in our experiments are specifically attributable to the disruption of *ahr-1* function rather than to confounding background mutations unique to one strain. This dual-strain approach enhances the rigor, reproducibility, and overall robustness of our findings.

Worms were maintained on nematode growth medium (NGM) plates seeded with *E. coli* OP50-1 at 24 °C under standard conditions.

To obtain synchronized populations, gravid hermaphrodites were treated with an alkaline hypochlorite solution to isolate embryos. Embryos were incubated on bacteria-free NGM plates for approximately 16 h. Subsequently, *E. coli* OP50-1 was seeded onto the plates, and worms were allowed to develop to the L4 stage. Synchronized L4 worms were transferred to fresh NGM plates supplemented with 12.5 mg/mL 5-fluorodeoxyuridine (FUdR) to prevent reproduction.

### 2.2. Preparation of Inactivated Bacterial Food

To prevent bacterial proliferation during dietary intervention experiments, *E. coli* OP50-1 was inactivated by kanamycin treatment. Briefly, *E. coli* OP50-1 cultures were grown overnight in LB medium containing 50 μg/mL kanamycin at 37 °C. Bacteria were then pelleted, washed twice with sterile M9 buffer, and seeded onto NGM plates containing 50 μg/mL kanamycin to ensure complete growth inhibition. Plates were dried overnight at room temperature before use.

### 2.3. Dietary Interventions

On day 2 of adulthood, synchronized worms were randomly assigned to one of three dietary regimens: AL: Worms were continuously maintained on plates seeded with kanamycin-inactivated *E. coli* OP50-1. IF: Worms were transferred between plates with or without inactivated *E. coli* OP50-1 every other day. FD: Worms were kept on NGM plates without any bacterial food from day 2 to day 8 or day 12 of adulthood, depending on the experimental group. This regimen reflects dietary deprivation as described in previous studies [21,22]. Importantly, extended periods of food deprivation (FD) have been shown to activate stress response pathways and, under specific conditions, promote increased longevity in *C. elegans* [21,22]. For IF, worms were alternately transferred between NGM plates seeded with kanamycin-inactivated *E. coli* OP50-1 and unseeded NGM plates every 48 h. For FD, we used a protocol of complete food deprivation [23], where animals were maintained on unseeded NGM plates without any bacterial food source for 8 or 12 days, depending on the specific experiment. Data for the IF group were collected after 48 h of food deprivation, and those for the AL and FD groups were collected at the same time points to ensure consistency. To minimize potential diurnal variations, all sample collection and imaging was performed between 9:00 and 10:00 a.m. All experiments were conducted at 24 °C, and each condition was replicated in at least three independent trials.

### 2.4. Tetramethylrhodamine (TMRE) Staining

Synchronized *C. elegans* were incubated on NGM plates supplemented with 100 nM TMRE for 16 h. Following staining, worms were washed with M9 buffer and allowed to recover for 1 h on fresh NGM plates seeded with *E. coli* OP50-1. Approximately 10–15 worms were then mounted on microscope slides in M9 buffer containing 10 mM sodium azide (NaN_3_) to immobilize them. Fluorescence imaging was performed using a Nikon Ts2R-FL fluorescence microscope under standardized conditions (e.g., consistent exposure time, light intensity, and magnification). For each worm, multiple fields were imaged to ensure comprehensive coverage. Fluorescence intensity was quantified using ImageJ (version 1.52v, National Institutes of Health, USA) by manually outlining individual worms and applying background subtraction. Statistical analysis was performed to compare fluorescence levels across experimental groups.

### 2.5. FITC-Dextran Uptake Assay

FITC-dextran staining was carried out as previously described [24], with modifications. Approximately 30–50 synchronized adult worms were transferred to 35 mm NGM plates containing *E. coli* OP50-1 and 20 μg/mL FITC-dextran (prepared from a 20 mg/mL stock). Worms were incubated for 14–15 h at 24 °C, and then washed with M9 buffer and allowed to crawl on fresh NGM plates seeded with *E. coli* OP50-1 for 1 h. After immobilization in M9 buffer with 10 mM NaN_3_, images were taken using a Nikon Ts2R-FL fluorescence microscope (Nikon Corporation, Tokyo, Japan) under consistent settings. Fluorescence intensity was analyzed using ImageJ, and average signal values were used for comparative analysis.

### 2.6. GFP-OP50 Bacterial Colonization Assay

GFP-labeled *E. coli* OP50 (GFP-OP50) were prepared by inoculating a single colony into LB broth (100 mL) containing 50 μg/mL ampicillin and culturing overnight at 37 °C with shaking (170 rpm). A 100 μL aliquot of the culture was spread on NGM plates (also containing 50 μg/mL ampicillin) and incubated overnight at 37 °C to allow bacterial growth.

Synchronized worms were washed with M9 buffer and transferred to GFP-OP50 plates for 24 h. Subsequently, they were moved to regular *E. coli* OP50-1-seeded NGM plates for 1 h to remove non-adherent bacteria. Worms were immobilized with 10 mM NaN_3_, and internal GFP-OP50 fluorescence in the intestine was visualized using the Nikon Ts2R-FL microscope. Image analysis was conducted using ImageJ to assess bacterial colonization levels.

### 2.7. Smurf Assay

To assess intestinal barrier integrity, the Smurf assay was performed using *C. elegans* immersed in a 1:1 mixture of S buffer and 5.0% (*w*/*v*) Erioglaucine disodium salt (blue food dye; Sigma-Aldrich, St. Louis, MO, USA) for 3 h [25].After staining, worms were washed three times with S buffer to remove residual dye, and then immobilized in M9 buffer containing 10 mM NaN_3_ on microscope slides.

Fluorescence images were acquired using a Nikon Ts2R-FL microscope under consistent imaging parameters. Intestinal integrity was evaluated by examining dye distribution: worms with dye confined to the intestinal lumen were classified as having an intact barrier, while those exhibiting dye leakage into the body cavity were considered to have compromised barrier function. The extent of intestinal damage was quantified by calculating the percentage of the dye-leaking intestinal length relative to the total intestinal length using ImageJ software.

### 2.8. MitoSOX Staining

Mitochondrial ROS levels were measured using MitoSOX^™^ Red (Thermo Fisher Scientific, Waltham, MA, USA), a fluorogenic dye selectively targeting mitochondrial superoxide. Synchronized young adult worms from each treatment group—AL, IF, and FD—were washed thoroughly with M9 buffer to remove bacteria. Worms were then incubated in 5 μM MitoSOX Red solution diluted in M9 buffer for 1 h at 20 °C in the dark with gentle agitation. After staining, worms were washed three times with fresh M9 buffer and transferred to agar pads for immediate imaging.

Image acquisition parameters were kept constant across all samples using a Nikon Ts2R-FL microscope (Nikon Corporation, Tokyo, Japan).MitoSOX fluorescence intensity was quantified using ImageJ. The mean fluorescence intensity per worm was calculated and used for statistical analysis.

### 2.9. Statistical Analysis

All statistical analyses were performed using GraphPad Prism 5.0. Data are expressed as mean ± standard error of the mean (SEM). Differences among multiple groups were evaluated using one-way analysis of variance (ANOVA), followed by Tukey’s multiple comparisons post hoc test. A *p*-value less than 0.05 was considered statistically significant.

## 3. Results

### 3.1. Fasting Reduces Intestinal Permeability in Wild-Type but Not in AHR Mutants

**Figure 1:**
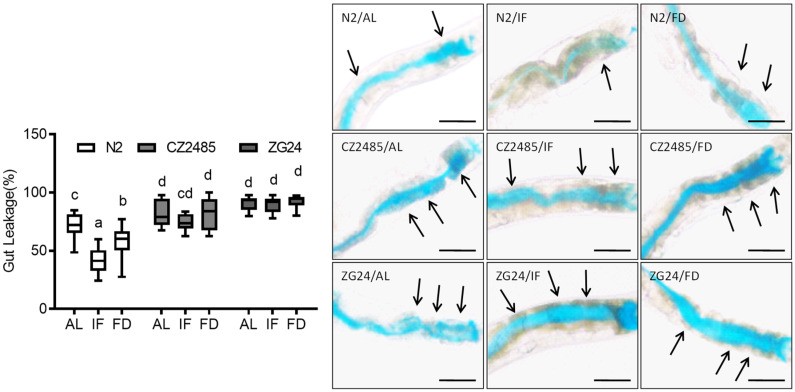
Effects of intermittent fasting and food deprivation on intestinal permeability in *C. elegans* on day 8, assessed using the Smurf assay. Wild-type (N2) and AHR mutant strains (CZ2485 and ZG24) were subjected to AL, IF, and FD. The blue dye leakage beyond the intestinal lumen indicates increased intestinal permeability. Arrows indicate regions of disrupted intestinal integrity. Data were obtained from at least three biologically independent experiments, with 15–20 worms analyzed per group per experiment. Different lowercase letters above the boxplots denote statistically significant differences among groups (*p* < 0.05, one-way ANOVA followed by Tukey’s post hoc test). Groups that share identical letters do not differ significantly, whereas groups with distinct letters are significantly different. Images originally acquired at 100× magnification. Scale bar = 25 μm.

To evaluate the effect of fasting on intestinal barrier integrity in *C. elegans*, we first employed the Smurf assay (Figure 1). In wild-type N2 worms, IF significantly reduced intestinal permeability compared to animals under AL. FD also decreased permeability relative to AL, although to a lesser extent than IF.

We next examined two AHR mutant strains, CZ2485 and ZG24. Under AL conditions, CZ2485 displayed permeability levels similar to those of wild-type N2. While IF induced a mild trend toward reduced permeability in CZ2485, FD unexpectedly increased permeability at approximately AL levels. In ZG24, both IF and FD failed to reduce permeability. The ZG24 group consistently exhibited high intestinal permeability across all dietary conditions, suggesting an AHR-dependent gut barrier protection in response to the dietary metabolic stress.

### 3.2. FITC-Dextran Assay Confirms Fasting-Induced Changes in Gut Permeability

**Figure 2:**
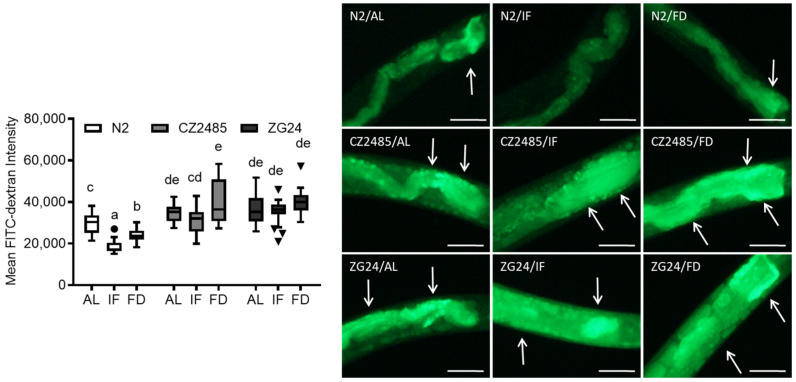
Intestinal permeability assessment via FITC-dextran uptake in *C. elegans* on day 12. Worms were exposed to FITC-dextran under AL, IF, and FD conditions. Fluorescence intensity was quantified in both wild-type and AHR mutant strains. Arrows indicate regions of increased FITC-dextran accumulation, reflecting disrupted intestinal integrity. Data were obtained from at least three biologically independent experiments, with 15–20 worms analyzed per group per experiment. Different lowercase letters above the boxplots denote statistically significant differences among groups (*p* < 0.05, one-way ANOVA followed by Tukey’s post hoc test). Data points outside of the boxplot indicate the outliers. Groups that share identical letters do not differ significantly, whereas groups with distinct letters are significantly different. Images originally acquired at 100× magnification. Scale bar = 25 μm.

To corroborate the Smurf assay results, we used FITC-dextran uptake to quantify intestinal permeability (Figure 2). In wild-type N2 worms, IF significantly reduced fluorescence intensity, indicating decreased permeability. FD also led to a significant reduction, though the protective effect was weaker than IF. These findings are consistent with the Smurf assay and reinforce the beneficial effect of fasting on gut barrier integrity.

In CZ2485 mutants, the baseline fluorescence in the AL group exceeded that of the N2 strain, suggesting a genetically compromised barrier function. Although the IF group exhibited a protective trend, the difference was not significant. However, FD even increased barrier permeability compared to AL. ZG24 mutants also exhibited a more compromised gut barrier than the N2 group: both IF and FD failed to counteract CR-induced protective action in the gut, with FD showing a pronounced exacerbation. These data indicate that the ZG24 group lacks the protective gut adaptation to fasting and is sensitive to nutrient stress.

### 3.3. GFP-OP50 Colonization Reveals Impaired Gut Defense in AHR Mutants Under Fasting

**Figure 3:**
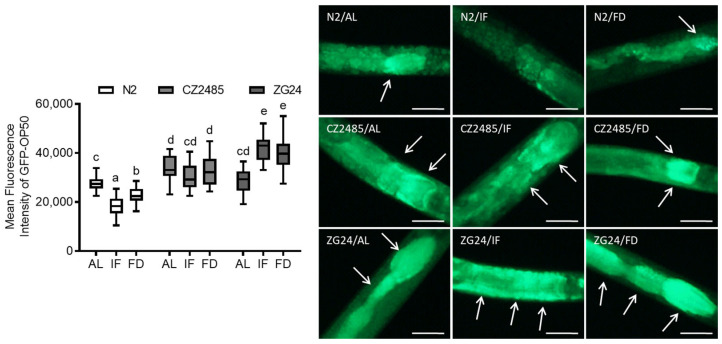
Quantification of intestinal bacterial colonization in *C. elegans* on day 12 using GFP-labeled *E. coli* OP50. Mean fluorescence intensity was measured to evaluate bacterial load under AL, IF, and FD conditions in both wild-type and AHR mutant strains. Arrows indicate regions of intestinal colonization by GFP-labeled *E. coli* OP50. Data were obtained from at least three biologically independent experiments, with 15–20 worms analyzed per group per experiment. Different lowercase letters above the boxplots denote statistically significant differences among groups (*p* < 0.05, one-way ANOVA followed by Tukey’s post hoc test). Groups that share identical letters do not differ significantly, whereas groups with distinct letters are significantly different. Images originally acquired at 100× magnification. Scale bar = 25 μm.

To further assess intestinal integrity and host defense, we measured gut colonization by GFP-labeled *E. coli* OP50 (Figure 3). In wild-type N2, IF and FD significantly reduced bacterial colonization relative to AL, which parallels results in the permeability assays and supports a fasting-induced enhancement of gut defense.

In AHR-mutant animals (CZ2485 and ZG24), the basal bacterial load across all dietary conditions was significantly higher than in N2, indicating a compromised intestinal defense. However, both IF and FD marginally counteracted bacterial colonization in the AHR mutants. Moreover, ZG24 showed a significant increase in bacterial colonization under both IF and FD conditions. Rather than improving gut defense, fasting appeared to aggravate bacterial susceptibility in this mutant, suggesting a critical role for AHR in regulating host–microbe interactions during nutritional stress.

### 3.4. Fasting Enhances Mitochondrial Function in Wild-Type but Not in AHR Mutants

**Figure 4:**
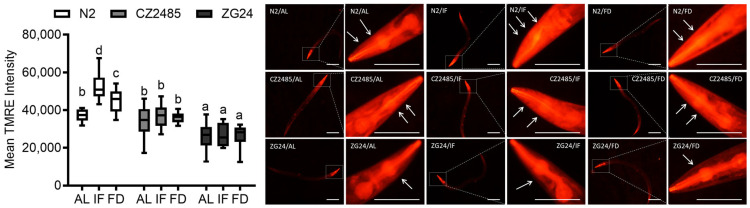
Mitochondrial membrane potential was measured by TMRE staining in *C*. *elegans* on day 12 under AL, IF, and FD conditions. Fluorescence intensity was quantified to evaluate mitochondrial activity in wild-type and AHR mutant strains. The **left panel** shows the full view of the worm, while the corresponding **right panel** presents a magnified image of the head region. Arrows indicate regions of strong TMRE fluorescence, reflecting high mitochondrial membrane potential. Data were obtained from at least three biologically independent experiments, with 15–20 worms analyzed per group per experiment. Different lowercase letters above the boxplots denote statistically significant differences among groups (*p* < 0.05, one-way ANOVA followed by Tukey’s post hoc test). Images originally acquired at 100× magnification. Scale bar = 100 μm.

To assess mitochondrial function, we measured mitochondrial membrane potential (ΔΨm) using TMRE staining in the worm gut (Figure 4). In wild-type N2, IF significantly enhanced mitochondrial activity, as evidenced by increased ΔΨm levels compared to AL. FD also improved ΔΨm, albeit to a lesser extent than IF.

In CZ2485, the basal ΔΨm under AL conditions was comparable to that of the N2 strain. However, in contrast to N2, neither IF nor FD significantly increased ΔΨm in the mutant, indicating a compromised mitochondrial response to fasting. ZG24 showed lower basal ΔΨm and similarly no response to either fasting condition. These data suggest that AHR deficiency compromises mitochondrial homeostasis and prevents fasting-induced mitochondrial enhancement.

### 3.5. Fasting Reduces Mitochondrial ROS in Wild-Type but Not in AHR Mutants

**Figure 5:**
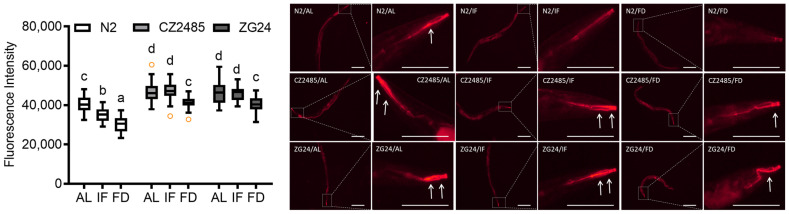
MitoSOX fluorescence imaging of mitochondrial ROS in *C*. *elegans* on day 12 under AL, IF, and FD conditions. The **left panel** shows the full view of the worm, while the corresponding **right panel** presents a magnified image of the head region. Arrows indicate regions of elevated MitoSOX fluorescence, representing increased mitochondrial ROS production. Data were obtained from at least three biologically independent experiments, with 15–20 worms analyzed per group per experiment. Data points outside of the boxplot indicate the outliers. Different lowercase letters above the boxplots denote statistically significant differences among groups (*p* < 0.05, one-way ANOVA followed by Tukey’s post hoc test). Images originally acquired at 100× magnification. Scale bar = 100 μm.

Mitochondrial ROS levels were evaluated using MitoSOX Red staining in wild-type N2 and AHR mutant strains (CZ2485 and ZG24) subjected to AL, IF, and FD (Figure 5). In N2 worms, MitoSOX fluorescence intensity followed the trend AL > IF > FD, indicating a progressive reduction in mitochondrial ROS with increasing dietary restriction. In contrast, both CZ2485 and ZG24 mutants exhibited a pattern of AL = IF > FD. Under FD conditions, mitochondrial ROS levels were reduced in both wild-type and AHR mutant strains. However, mitochondrial ROS levels in the mutants remained higher than those in wild-type worms, indicating that AHR plays a role in maintaining the mitochondrial redox balance under CR conditions.

## 4. Discussion

In this study, we demonstrate that IF exerts protective effects on intestinal barrier integrity and mitochondrial function in *C. elegans*. Using multiple complementary assays—including the Smurf assay, FITC-dextran uptake, GFP-OP50 colonization, and TMRE staining—we show that IF reduces intestinal permeability, limits bacterial overgrowth, and enhances mitochondrial membrane potential and ROS management in wild-type animals. These findings are consistent with previous reports in mammalian models linking dietary restriction to improved intestinal resilience and metabolic efficiency under stress conditions [8,10,24].

Importantly, our study reveals that the AHR plays a critical role in mediating these fasting-induced benefits. While the wild-type worms responded robustly to IF, AHR mutants (CZ2485 and ZG24) displayed impaired gut barrier adaptation and failed to exhibit mitochondrial improvements under the same dietary conditions. Particularly in ZG24, fasting led to notable increases in intestinal permeability and bacterial colonization, suggesting a loss of protective fasting responses. These observations indicate that AHR signaling is essential for the physiological adaptation to nutrient deprivation [16,17,18].

AHR is a ligand-activated transcription factor traditionally known for its role in xenobiotic detoxification. However, accumulating evidence indicates that AHR also modulates redox homeostasis, immune responses, and epithelial barrier function—all of which are central to maintaining gut health [15,25]. In mammalian systems, AHR activation has been shown to upregulate antioxidant genes such as Nrf2, SOD, and Gpx, as well as to regulate tight junction proteins in the intestinal epithelium [26,27]. Our findings suggest that similar protective mechanisms may be conserved in *C. elegans* and that AHR is required to transduce fasting signals into redox- and barrier-protective responses.

The present study demonstrates that mitochondrial function is tightly linked to both redox balance and epithelial integrity. The observed enhancement of ΔΨm in fasted wild-type animals suggests a shift toward more efficient energy metabolism or mitochondrial biogenesis. In contrast, AHR mutants failed to respond to fasting with increased ΔΨm and ROS production, implying potential mitochondrial dysregulation. This aligns with prior studies reporting that AHR deficiency leads to mitochondrial fragmentation, impaired oxidative phosphorylation, or increased ROS in various model organisms [28,29,30].

MitoSOX staining revealed that mitochondrial ROS levels were significantly reduced by IF and FD in wild-type (N2) worms, following the trend AL > IF > FD. This suggests that dietary restriction lowers oxidative stress, possibly by reducing mitochondrial metabolic load [31,32]. In contrast, AHR mutants (CZ2485 and ZG24) exhibited persistently high ROS levels across all conditions, with IF showing marginal protective effects (AL = IF > FD), indicating that AHR is required for fasting-induced redox protection in a calorie-dependent manner [15]. These redox patterns were consistent with other observations: lower mitochondrial ROS in wild-type worms paralleled improved gut barrier integrity (Smurf, FITC-dextran), reduced bacterial colonization (GFP-OP50), and enhanced mitochondrial membrane potential (TMRE). Conversely, high mitochondrial ROS in mutants coincided with impaired barrier function and mitochondrial defects [1]. Together, these results suggest that AHR links nutritional cues to redox regulation and epithelial homeostasis during fasting.

Furthermore, the differential effects of IF and FD underscore the importance of dietary pattern and duration. While IF induced consistent protective responses in wild-type worms, FD showed more modest and sometimes variable effects, particularly in mutants. These findings support the notion that IF—not simply nutrient absence—is a structured hormetic stimulus that requires intact transcriptional regulation to confer benefits [33,34].

Taken together, our results support a model in which CR promotes intestinal and mitochondrial health through AHR-dependent pathways. Fasting enhanced redox balance, suppressed microbial overgrowth, and subsequently maintained epithelial barrier integrity. The failure of AHR mutants to mount such beneficial responses highlights the indispensable role of AHR in linking metabolic stress to gut health. Given the evolutionary conservation of AHR and its emerging role in regulating metabolic stress in the gut, these findings may have broader implications, particularly regarding dietary interventions for managing inflammatory and metabolic distress in humans. Future studies are needed to identify dietary AHR ligands that could serve as metabolic regulators in the gut, mimicking the beneficial effects of dietary restriction [15,25,35].

## 5. Conclusions

This study provides new insights into the genetic regulation of intestinal barrier function in response to dietary interventions. Intermittent fasting enhances gut integrity, limits microbial colonization, and improves mitochondrial function and redox management in *C. elegans*, but these benefits require functional AHR signaling. The inability of AHR mutants to respond to fasting reveals a previously unrecognized role for AHR in linking nutrient availability to intestinal homeostasis and mitochondrial health. Our results underscore the importance of gene–diet interactions in maintaining gut health and highlight AHR as a potential therapeutic target for dietary modulation strategies aimed at preventing or mitigating intestinal disorders. Further investigation into the molecular pathways regulated by AHR will be crucial for developing precision nutrition interventions to enhance intestinal resilience and organismal longevity.

## Data Availability

Data used in the present study are available from the corresponding author on reasonable request.

## Abbreviations

The following abbreviations are used in this manuscript:

*C. elegans*	*Caenorhabditis elegans*
AL	Ad libitum
IF	Intermittent fasting
FD	Complete food deprivation
AHR	Aryl hydrocarbon receptor
